# Modified mesenchymal stromal cells by in vitro transcribed mRNA: a therapeutic strategy for hepatocellular carcinoma

**DOI:** 10.1186/s13287-024-03806-0

**Published:** 2024-07-11

**Authors:** María José Cantero, Barbara Bueloni, Lucrecia Gonzalez Llamazares, Esteban Fiore, Lucia Lameroli, Catalina Atorrasagasti, Guillermo Mazzolini, Mariana Malvicini, Juan Bayo, Mariana G. García

**Affiliations:** 1 Experimental Hepatology and Gene Therapy Program, Instituto de Investigaciones en Medicina Traslacional (IIMT), Universidad Austral - Consejo Nacional de Investigaciones Científicas y Técnicas (CONICET), Buenos Aires, Argentina; 2 Cancer Immunobiology Laboratory, IIMT, Universidad Austral - CONICET, Buenos Aires, Argentina

**Keywords:** Mesenchymal stromal cell, Granulocyte-macrophage colony-stimulating factor, In vitro transcribed mRNA, Immunogenic cell death, Immunotherapy, Hepatocellular carcinoma

## Abstract

**Background:**

Mesenchymal stromal cells (MSCs) tropism for tumours allows their use as carriers of antitumoural factors and in vitro transcribed mRNA (IVT mRNA) is a promising tool for effective transient expression without insertional mutagenesis risk. Granulocyte-macrophage colony-stimulating factor (GM-CSF) is a cytokine with antitumor properties by stimulating the specific immune response. The aim of this work was to generate modified MSCs by IVT mRNA transfection to overexpress GM-CSF and determine their therapeutic effect alone or in combination with doxorubicin (Dox) in a murine model of hepatocellular carcinoma (HCC).

**Methods:**

DsRed or GM-CSF IVT mRNAs were generated from a cDNA template designed with specific primers followed by reverse transcription. Lipofectamine was used to transfect MSCs with DsRed (MSC/DsRed) or GM-CSF IVT mRNA (MSC/GM-CSF). Gene expression and cell surface markers were determined by flow cytometry. GM-CSF secretion was determined by ELISA. For in vitro experiments, the J774 macrophage line and bone marrow monocytes from mice were used to test GM-CSF function. An HCC model was developed by subcutaneous inoculation (s.c.) of Hepa129 cells into C3H/HeN mice. After s.c. injection of MSC/GM-CSF, Dox, or their combination, tumour size and mouse survival were evaluated. Tumour samples were collected for mRNA analysis and flow cytometry.

**Results:**

DsRed expression by MSCs was observed from 2 h to 15 days after IVT mRNA transfection. Tumour growth remained unaltered after the administration of DsRed-expressing MSCs in a murine model of HCC and MSCs expressing GM-CSF maintained their phenotypic characteristic and migration capability. GM-CSF secreted by modified MSCs induced the differentiation of murine monocytes to dendritic cells and promoted a proinflammatory phenotype in the J774 macrophage cell line. In vivo, MSC/GM-CSF in combination with Dox strongly reduced HCC tumour growth in C3H/HeN mice and extended mouse survival in comparison with individual treatments. In addition, the tumours in the MSC/GM-CSF + Dox treated group exhibited elevated expression of proinflammatory genes and increased infiltration of CD8 + T cells and macrophages.

**Conclusions:**

Our results showed that IVT mRNA transfection is a suitable strategy for obtaining modified MSCs for therapeutic purposes. MSC/GM-CSF in combination with low doses of Dox led to a synergistic effect by increasing the proinflammatory tumour microenvironment, enhancing the antitumoural response in HCC.

**Supplementary Information:**

The online version contains supplementary material available at 10.1186/s13287-024-03806-0.

## Introduction

Mesenchymal stromal cells (also known as mesenchymal stem cells; MSCs) are immune-privileged multipotent progenitors [1] that can modulate immune and inflammatory responses [2] and migrate to injury sites [3]. Initially, isolated from the bone marrow (BM-MSCs), MSCs can be expanded from almost all tissues, with adipose tissue or the umbilical cord being the most common sources. In the latter case, MSCs can be isolated from Wharton’s jelly (WJ-MSCs), perivascular areas (HUCPVCs), or umbilical cord blood (CBMSCs) [4, 5]. Accumulating evidence indicates that MSCs transplanted under different pathological conditions can home to sites of tissue injury and induce the recruitment of endogenous cells, tissue remodelling, and anti-inflammatory activities [6]. On the other hand, it is known that MSCs can migrate and engraft into tumours, and it is generally believed that this property is influenced by factors produced by tumour cells. In fact, we have previously demonstrated that BM-MSCs and HUCPVCs can home hepatocellular carcinoma (HCC) tumours and that this ability is enhanced by autocrine motility factor (AMF) [7]. Because of all these unique biological properties, MSCs have attracted increased attention, especially in cell therapy. It has even been demonstrated that MSCs from certain sources, such as HUCPVC, have enhanced antitumourigenic properties in certain types of tumours [8–11]. The principal interest within the therapeutic applications of MSCs derives from their use as carriers of antitumoural genes for the treatment of several cancer types [12]. In this context, MSCs can be engineered to deliver specific genes, and the use of viral vectors was initially the preferred method for enabling sustained exogenous gene expression by MSCs [13–16]. However, this approach has significant drawbacks, including the risk of insertional mutagenesis or viral integration into the MSC genome and an increase in immunogenicity [17, 18].

To replace viral vectors, several transgene-free technologies for transduction have recently been developed. In particular, in vitro transcribed messenger RNA (IVT mRNA) is a nonviral RNA-based technology for gene expression that can efficiently overexpress a target gene by exploiting the cell’s own cellular translation machinery without the risk of insertional mutagenesis. In contrast to viral gene therapy vectors, synthetic mRNAs do not enter the nucleus; therefore, integration into the genome and the risk of insertional mutagenesis and oncogenesis are avoided [19]. Another advantage of IVT mRNA is that the expression level of the target gene can be adjusted and controlled [20]. For IVT mRNA generation, different modifications can be introduced during transcription for efficient translation and stability of synthetic mRNAs. To increase mRNA stability, an anti-reverse cap analogue (ARCA, 3´-O-Me-m7G(5´)ppp(5´)G) on the 5’-end is added [21]. Considering that exposure to nonself nucleic acids activates the mammalian innate immune system, the incorporation of modified nucleosides such as 5-methylcytidine and pseudouridine within mRNA molecules reduces their immunogenicity and stabilizes them against RNase cleavage [22–24]. Taken together, these findings suggest that all these modifications of IVT mRNA improve transfection efficiency and translation via the intracellular machinery, as demonstrated by improved cytosolic persistence and protein expression. Therefore, IVT mRNA constitutes a safer and more efficacious method for achieving gene expression in clinical applications. This RNA-based technology has been extensively used to reprogram somatic cells into pluripotent stem cells [25], and particularly in cancer immunotherapy, to produce chimeric antigen receptor (CAR)-engineered T cells [26]. However, MSC modification by IVT mRNA has been poorly explored.

Hepatocellular carcinoma (HCC) is the sixth most common cancer worldwide and the third leading cause of cancer-related deaths [27]. It is the most common type of primary liver tumour and generally arises from chronic liver diseases caused by viral hepatitis, metabolic syndrome or alcohol abuse. The overall prognosis of patients with HCC is poor; only a small fraction of patients who are diagnosed at early stages of the disease are eligible for curative therapies such as liver resection, transplantation or interventional ablation. Since most patients receive only palliative treatments, new therapeutic approaches are needed [28]. HCC is characterized by an immunosuppressive microenvironment; therefore, efforts are focused on overcoming this unfavourable milieu and generating a potent immune response [29]. Several approaches, such as dendritic cell-based vaccines [30], checkpoint-blocking therapies [31, 32], and adoptive T-cell transfer, have been tested but have shown low efficacy as monotherapy options in treating HCC [33, 34]. The limited efficacy of these treatments is probably due to a variety of factors, such as the anti-inflammatory immune environment in the liver, which aims to tolerate foreign antigens; the low immunogenicity of malignant cells; the defective antigen cross-presentation by dendritic cells (DCs); and the exhaustion of cytotoxic T cells, among others [35]. Therefore, our work focused on the combination of two therapeutic approaches: first, stimulating antigen-presenting cells (APCs) with IVT mRNA-modified MSCs expressing granulocyte-macrophage colony-stimulating factor (MSC/GM-CSF); second, enhancing the antitumoural effect of immunogenic cell death (ICD) induced with doxorubicin (Dox). We found that IVT mRNA-modified MSCs is a very effective method for expressing and secreting proteins, improving the therapeutic potential of MSCs. Furthermore, we demonstrated a synergistic interaction between treatments of our combined therapeutic approach in an HCC tumour model. In conclusion, IVT mRNA transfection is a suitable strategy for obtaining modified MSCs for therapeutic purposes.

## Materials and methods

### Mesenchymal stromal cell (MSC) culture

MSCs were isolated from human umbilical cords obtained from healthy donors at the Hospital Universitario Austral (Pilar, Buenos Aires, Argentina) as we previously described (Protocol approval #16–038) [7]. In brief, umbilical cords were dissected, and vessels with their surrounding Warthon’s jelly were pulled out. Then, perivascular mesenchymal tissue was removed from the vessels and mechanically disrupted. Minced fragments were plated in complete αMEM/20% foetal bovine serum (FBS; Internegocios S.A., Argentina). After 7 days of incubation, the nonadherent cells and minced fragments were removed, and the adherent MSCs were cultured and used for different experiments at passages 5 to 7.

### IVT design and MSC transfection

**Amplification of plasmid inserts and addition of poly(T) tail by polymerase chain reaction (PCR).** The pCR3.1 vector containing the coding sequence (CDS) of murine GM-CSF (mGM-CSF; Addgene, cat#74465) or the pAAV-IRES-DsRed vector containing the CDS of DsRed (gift from Dr. Rodolfo Goya, School of Medicine, National University of La Plata, Argentina) were used as templates. To amplify the CDS of mGM-CSF and DsRed, the Hotstar HiFidelity Polymerase Kit (Qiagen, Hilden, Germany) was used in accordance with the manufacturer’s instructions. For PCR, 100 ng of plasmid DNA, 0.7 µM forward primer and 0.7 µM reverse primer (Supplementary Table 1) were used. PCR was performed using the following cycling protocol: initial activation at 95 °C for 5 min; 30 cycles of denaturation at 95 °C for 45 s, annealing at 57 °C for 1 min, extension at 72 °C for 1 min; and a final extension at 72 °C for 10 min. After DNA amplification, the PCR products were purified using a PCR purification kit (Dongsheng Biotech) and eluted in 2 × 20 µl of nuclease-free water. The quality and purity of the DNA were assessed via 1% agarose gel electrophoresis.

**IVT mRNA.** The IVT of the DNA into mRNA was performed using a MEGAscript^®^ T7 Kit (Life Technologies, Darmstadt, Germany) according to the manufacturer’s instructions. The IVT reaction mixture contained 7.5 mM ATP, 1.875 mM GTP (both from MEGAscript T7 Kit), 7.5 mM Me-CTP (TriLink BioTechnologies, San Diego, USA), 7.5 mM Pseudo-UTP (TriLink BioTechnologies), 2.5 mM ARCA (3´-O-Me-m7G (5´) ppp(5´)G RNA cap structure analog) (New England Biolabs, Frankfurt am Main, Germany), 40 U RiboLock RNase inhibitor (Thermo Fisher Scientific, Waltham, USA), 1 µg PCR product, 1x reaction buffer and 1x T7 RNA polymerase enzyme. The mixture was incubated for 4 h at 37 °C, after which 1 µl of TURBO DNase (from MEGAscript T7 Kit) was added to the IVT reaction mixture and subsequently incubated for 15 min at 37 °C to remove the template DNA. Then, dephosphorylation was performed with 10 U of Antarctic phosphatase (New England Biolabs) at 37 °C for 30 min. After the incubation, the mRNA was purified using a General RNA Extraction Kit (Dongsheng Biotech) and eluted in 2 × 10 µl of nuclease-free water. The concentration was measured using a NanoDrop spectrophotometer. The quality and purity of the synthesized and modified mRNAs were confirmed via 1% agarose gel electrophoresis. The modified mRNA was stored at − 80 °C and used for transfections.

**Transfection of MSCs with IVT mRNA.** For transfection, 25 µl of Opti-MEM, 0.5 µl of Lipofectamine 2000 (Thermo Fisher Scientific) and 0.2–0.4 µg of IVT mRNA from DsRed or GM-CSF were mixed and incubated for 20 min at room temperature to induce lipoplexes formation. MSCs were trypsinized, resuspended in Opti-MEM/10% FBS, and 4 × 10^4^ MSCs were incubated with the transfection complexes for 3 h at 37 °C in 5% CO_2_ with gentle shaking. After incubation, MSCs transfected with IVT mRNA from DsRed (MSC/DsRed) or GM-CSF (MSC/GM-CSF) were seeded in the wells of 48-well plates supplemented with 150 µl of complete medium αMEM/10% FBS.

### In vitro migration

In vitro migration of MSCs was assessed using a 48-Transwell microchemotaxis Boyden Chamber unit (Neuroprobe, Inc.) as previously described [36]. MSCs transfected with IVT mRNA o untransfected (1.2 × 10^3^ cells/well) were placed in the upper chamber, and α-MEM or tumour-conditioned medium (TCM) was added to the lower chamber of the Transwell unit.

### Cell viability assay

Cell viability was determined using an MTT (3-(4,5-dimethylthiazol-2-yl)-2,5-diphenyltetrazolium bromide) assay according to the manufacturer’s instructions (Invitrogen).

### Cell lines

Hepa129 cells (HCC cells syngeneic with C3H/HeN mice) were kindly provided by Dr. Volker Schmitz (Bonn University, Germany). The murine macrophage line J774 was used. Both cell lines were grown in complete RPMI 1640 (2 mM glutamine, 100 U/ml penicillin, 100 mg/ml streptomycin) supplemented with 10% heat-inactivated foetal bovine serum (FBS).

### [^3^H]-Thymidine uptake splenocyte proliferation assay

Lymphocyte proliferation was assessed in vitro by stimulation with concanavalin-A (ConA) as a positive control for proliferation, conditioned media (CM) from MSCs/DsRed, CM from MSCs/GM-CSF or RPMI (control) for 3 days, followed by a pulse of [^3^H]-thymidine 18 h before the end of the experiment. [^3^H]-thymidine incorporation was measured in a scintillation counter.

### Generation of bone marrowderived dendritic cells (DCs)

DCs were generated from murine bone marrow cells as described previously [37]. Briefly, the bone marrow of C3H/HeN mice was obtained from femurs and tibias and subjected to mechanical disruption. Cell suspensions were generated and cultured with complete RPMI 1640 supplemented with 10% FBS, GM-CSF (20 ng/ml; PeproTech, Germany) or conditioned medium from MSC/GM-CSF (CM MSC/GM-CSF). On days 3 and 5, the medium was removed and replaced with fresh RPMI 1640. On day 7, cells in suspension were collected (DCs) and used for the experiments.

### Animals and in vivo experiments

Four- to six-week-old male C3H/HeN or Balb/C mice were purchased from the School of Medicine (Universidad de Buenos Aires, Argentina). Animals were maintained at our animal resources facilities in accordance with the experimental ethical committee and the NIH guidelines on the ethical use of animals. The experimental protocol (No. 2019-15) was approved by the Animal Care Committee of the School of Biomedical Sciences, Universidad Austral, based on the essential points of the ARRIVE guidelines.

To assess in vivo tumour growth syngeneic immunocompetent mouse models were used. Six- to eight-week-old C3H/HeN or Balb/C mice were subcutaneously (s.c.) injected into the right flank with 1 × 10^6^ Hepa129 cells or 5 × 10^5^ CT-26 cells, respectively. Tumour volume was measured using a calliper 3 times per week. When the tumour volume reached ~60 mm^3^, mice were divided into groups (*n* = 6/group) and received saline (control) or 2 × 10^5^ MSC/GM-CSF. To evaluate the therapeutic potential of the combined treatment, the C3H/HeN model was used: mice were divided into groups (*n* = 7/group) and received saline (control), 5 mg/kg doxorubicin (Dox), and the next day 2 × 10^5^ MSC/GM-CSF or both treatments (MSC/GM-CSF + Dox). On day 7, the mice were sacrificed, and tumours samples were collected for cytometry, RT‒PCR or cytokine assays. All treatments were previously randomized in each experiment for both murine models. For euthanasia, animals were deeply anaesthetized in a CO_2_ chamber and sacrificed by cervical dislocation.

### Analysis of in vivo interactions between treatments

To calculate the dose enhancement factor to reach the tumour a volume of 300 mm^3^ (DEF300), the following formula was used: (time to reach a tumour volume of 300 mm^3^ by the combination treatment - time to reach 300 mm^3^ by individual treatment) / (time to reach 300 mm^3^ by individual treatment - time to reach 300 mm^3^ in control).

To evaluate whether there is a synergistic effect between treatments, the fractional product method (FTV) was used [38] in an HCC model. The following formula was used to calculate the FTV: (experimental mean tumour volume) / (control mean tumour volume), considering the day after treatment onset; (MSC/GM mean FTV) x (Dox mean FTV); R = [Expected FTV/Observed FTV].

### RNA isolation and quantitative PCR analysis

Total RNA was isolated from the samples using TRIzol Reagent (Sigma-Aldrich Co.) and total RNA (2 µg) was reverse transcribed (RT-qPCR) with 200 U of SuperScript II Reverse Transcriptase (Invitrogen) using 500 ng of oligo (dT) primers. cDNAs were subjected to real-time polymerase chain reaction (qPCR). The mRNA levels of interleukin-1 beta (IL-1β), tumour necrosis factor alpha (TNF-α), CD8, F4/80, CD11c, tapasin and ERp57 were quantified using SYBR Green (Invitrogen). Amplifications were carried out using a cycle of 95 °C for 10 min and 40 cycles of 95 °C for 30 s, 56 °C for 30 s, and 72 °C for 1 min. At the end of the PCR, the temperature was increased from 60 to 95 °C at a rate of 2 °C/min, and fluorescence was measured every 15 s to construct the melting curve. The values were normalized to the levels of the glyceraldehyde-3-phosphate dehydrogenase transcript (GAPDH), which was used as a housekeeping gene. The expression of GAPDH was not significantly different between the groups. The data were processed by the ΔΔCt method, and the relative amount of the PCR product amplified from saline (control) tumour tissue samples or media in the case of cell culture (RPMI) was set as 1. A no template control (NTC) was used in every assay, and all determinations were performed in triplicate for each animal or cell culture well. The list of primers used is provided in Supplementary Table 2.

### GM-CSF measurement

The levels of GM-CSF were determined using an ELISA kit (R&D Systems). All procedures were performed following the manufacturer’s instructions. The data were derived from three independent experiments.

### Surface marker analysis by flow cytometry

Single-cell suspensions from MSC/GM-CSF or untransfected MSCs were stained with antibodies against CD34, CD44, CD80, CD86, CD90 and MHCII (BD Biosciences). The characterization of DCs was performed with antibodies against CD11c, CD86 and MHCII. Single-cell suspensions from tumour samples pretreated with collagenase (Sigma-Aldrich, USA) were stained with antibodies against F4/80, MHCII, CD3 and CD8 (BD Biosciences). In all the cases the antibodies were incubated for 45 min at 4 °C. After washing, cell surface markers were measured on a BD Accuri™ C6 Plus (BD Biosciences), and the results were analysed with BD Accuri C6 plus software.

### Statistical analysis

All the experiments were repeated at least 3 times on different occasions. The values are expressed as the mean ± standard error of the mean (SEM) as indicated for each experiment. Two-way ANOVA was used to evaluate the statistical differences between groups. Mouse survival was analysed by Kaplan-Meier curves. A *p* value < 0.05 was considered to indicate statistical significance. Prism software (GraphPad, San Diego, CA, USA) was used for the statistical analysis.

## Results

### Transfection with IVT mRNA is a suitable strategy for obtaining modified MSCs

**Fig. 1:**
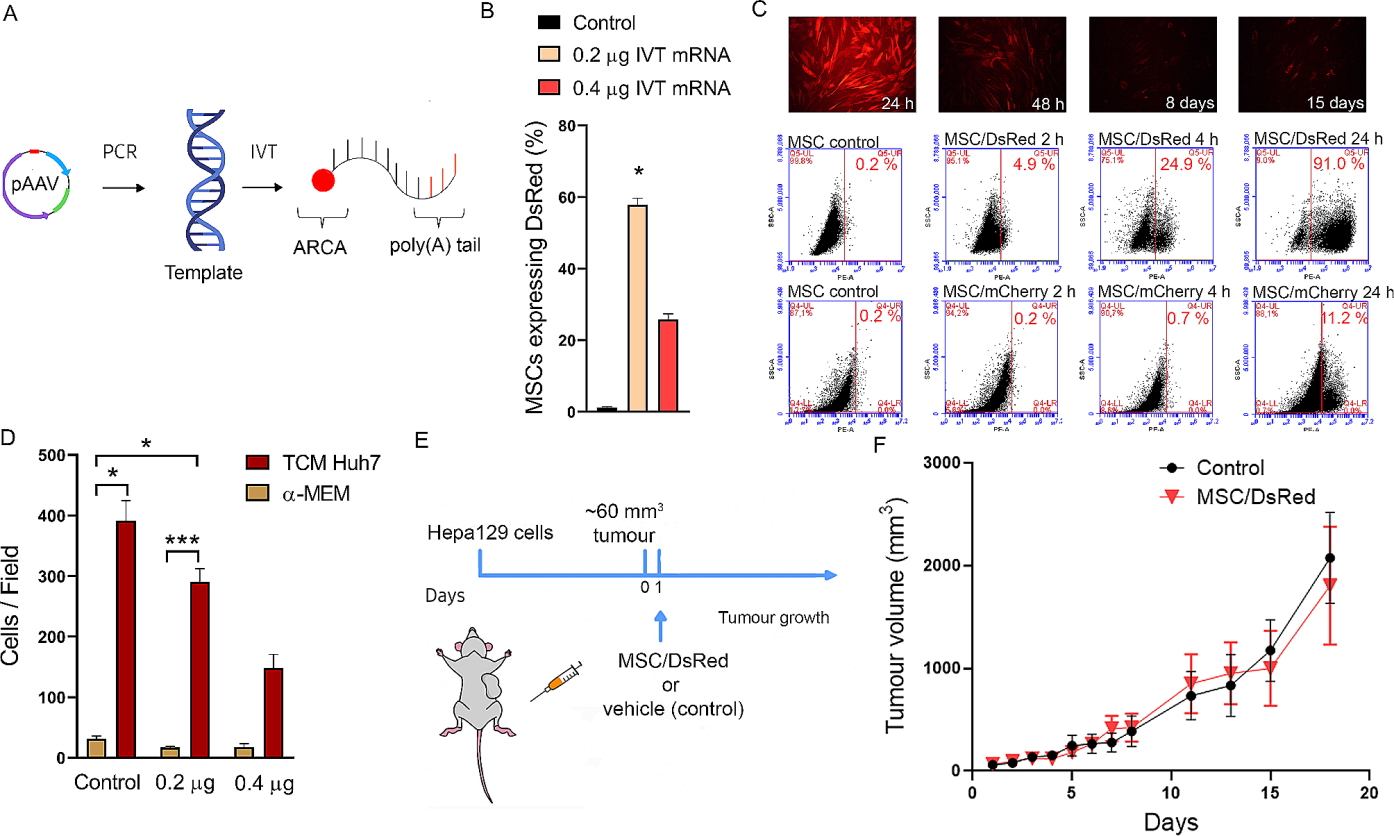
Transfection with IVT mRNA is a suitable strategy for obtaining engineered MSCs. **A** Scheme of IVT mRNA design. The coding sequence (CDS) of DsRed was amplified via PCR from the pAAV plasmid to generate the template. In vitro transcription (IVT) requires a mixture of nucleosides triphosphate (ATP, GTP, methylcytidine-5’-triphosphate (MeCTP), pseudouridine-5’-triphosphate (PseudoUTP)) and 3´-O-Me-m7G (5´) ppp(5´)G RNA cap structure analog (ARCA) to obtain a complete IVT mRNA. **B** Expression of DsRed was analysed by flow cytometry in MSCs transfected with different amounts of IVT mRNA (0.2–0.4 µg). Dunn’s multiple comparisons test, *p < 0.05 vs. control. **C** Representative images by fluorescence microscopy of MSCs transfected with 0.2 µg of IVT mRNA of DsRed at 24 h, 48 h, 8 days and 15 days (top panel). Kinetics of IVT mRNA expression determined by flow cytometry (middle panel) in comparison with the plasmid transfection method (bottom panel) at 2 h, 4 h and 24 h. **D** Untransfected MSCs (control) or MSC/DsRed transfected with 0.2–0.4 µg of IVT mRNA were evaluated for their ability to migrate toward tumour conditioned medium derived from Huh7 cells (TCM Huh7) or α-MEM in a modified Boyden chamber. Dunn’s multiple comparisons test, *p < 0.05 and **p < 0.01 vs. α-MEM (basal migration). **E** Experimental model: C3H/HeN mice were subcutaneously (s.c.) injected with 1 × 10^6^ syngeneic Hepa129 cells, and when the tumour reached ~60 mm^3^ (day 0), 2 × 10^5^ MSC/DsRed or saline (control) was s.c. injected (day 1). **F** In vivo tumour growth of Hepa129 tumour-bearing mice with vehicle (control) or MSC/DsRed. Two-way ANOVA and Sidak’s multiple comparisons test

To evaluate whether transfection with IVT mRNA is a suitable method for inducing protein expression in MSCs, we produced DsRed IVT mRNA following the strategy shown in Fig. 1A. First, the promoter of T7 RNA polymerase and the coding sequence (CDS) of DsRed were amplified from the pAAV vector by PCR using specific primers that included a poly(T) tail in the reverse primer. Second, IVT mRNA was generated using the PCR product as a template with a nucleoside triphosphate/cap synthetic mixture for increased stability and transcription efficiency and with T7 RNA polymerase. This strategy allows us to obtain IVT mRNA at high concentrations (up to 1.5 µg/µl). Then, we determined the expression efficiency of two different amounts of DsRed IVT mRNA (0.2–0.4 µg) by transfecting 4 × 10^4^ MSCs with Lipofectamine. We found that MSCs transfected with 0.2 µg of DsRed IVT mRNA expressed higher levels of DsRed (Fig. 1B). Next, to gain insight into the expression kinetics of this engineering method in MSCs, DsRed expression was analysed by both flow cytometry and fluorescence microscopy. Remarkably, we found that modified MSCs expressed DsRed as quickly as 2 h, with a maximum at 24 h, maintaining DsRed expression for up to 15 days (Fig. 1C top and middle panels). We analysed the protein expression obtained by the IVT mRNA method in comparison with that obtained with the use of a plasmid containing the mCherry sequence. We found that MSCs transfected with a plasmid needed additional time to express exogenous proteins (Fig. 1C bottom). These results indicated that transfection using IVT mRNA is effective for rapid expression and that the effect is maintained for a period of time. Since the interest in the use of MSCs as a vehicle for therapeutic factor delivery is based, at least in part, on their tropism for tumours and damaged tissues, we tested whether transfection with IVT mRNA could alter their migratory ability. We observed that after 4 × 10^4^ MSCs were transfected with 0.4 µg of IVT mRNA, their migration toward conditioned media (CM) derived from an HCC cell line (Huh7) was significantly lower than that of untransfected MSCs (Fig. 1D). However, the migration of MSCs transfected with 0.2 µg / 4 × 10^4^ cells was similar to that of untransfected cells (control). Considering that modified MSCs could be useful tools for in vivo cancer treatment, we used 0.2 µg / 4 × 10^4^ MSCs in the following experiments. Next, we tested whether the administration of MSCs/DsRed to mice bearing a syngeneic HCC cell line (Hepa129) affects tumour growth according to the scheme in Fig. 1E. As shown in Fig. 1F, there was no difference in tumour growth between mice that received MSCs/DsRed or vehicle (control). Taken together, these results suggest that the use of MSCs modified with IVT mRNA could lead to a new therapeutic strategy for the delivery of protein.

### Modified MSCs to overexpress GM-CSF by IVT mRNA transfection have therapeutic potential for tumour treatment

**Fig. 2:**
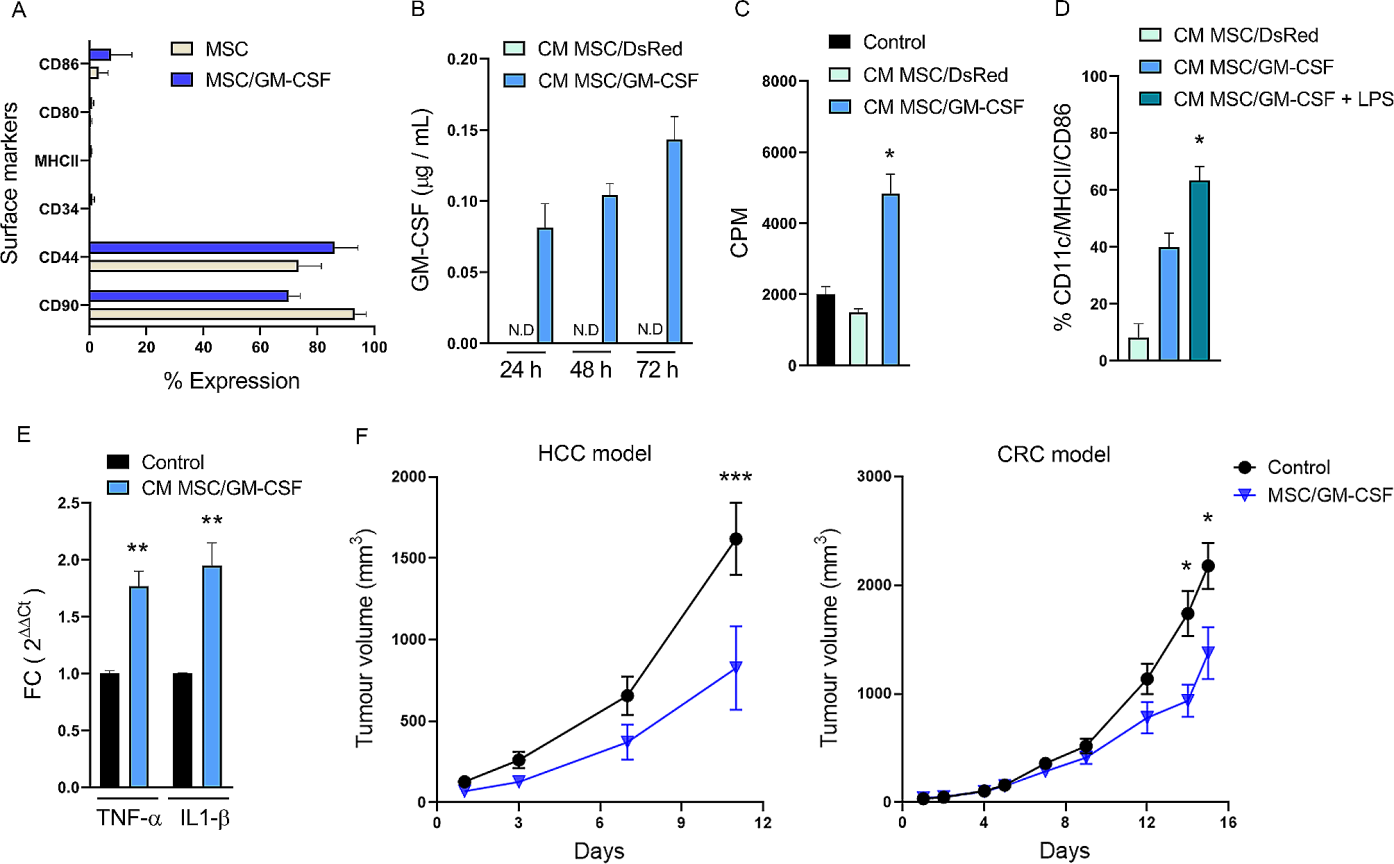
MSCs engineered to overexpress GM-CSF by IVT mRNA. **A** Percentage of expression of surface markers in untransfected MSCs (MSC) and MSCs transfected with 0.2 µg IVT mRNA / 4 × 10^4^ cells (MSC/GM-CSF) analysed by flow cytometry. Sidak’s multiple comparisons test was not significant for any marker. **B** GM-CSF production in the conditioned media of MSC/DsRed (CM MSC/DsRed) or MSC/GM-CSF (CM MSC/GM-CSF) analysed by ELISA. **C** In vitro proliferation of murine splenocytes cultured with ConA plus CM MSC/GM-CSF, CM MSC/DsRed or RPMI (control). Cell proliferation was evaluated by [^3^H]-thymidine incorporation assay and results are expressed as counts per minute (CPM). Dunn’s multiple comparisons test, *p < 0.05 vs. control. **D** Analysis of DCs surface markers (CD11c, MHCII and CD86) in murine bone marrow cells cultured with CM MSC/DsRed, CM MSC/GM-CSF or CM MSC/GM-CSF plus LPS. Dunn’s multiple comparisons test, *p < 0.05 vs. CM MSC/DsRed. **E** mRNA expression of TNF-α and IL-1β in J774 cells stimulated with CM from MSC/GM-CSF or RPMI as control determined by RT-qPCR. Mann-Whitney test, **p < 0,001 vs. control. **F** Tumour growth in the HCC mouse model (left) or colorectal carcinoma murine model (right). When the tumours reached ~60 mm^3^ peritumoral injection of 2 × 10^5^ MSC/GM-CSF or PBS (control) was administered. Two-way ANOVA and Sidak’s comparison test, *p < 0.05 and ***p < 0.001 vs. control

As we showed, modified MSCs with IVT mRNAs could be used for the delivery of therapeutic factors. Therefore, we evaluated this approach via the use of modified MSCs with IVT GM-CSF mRNA (MSC/GM-CSF) to stimulate an antitumour immune response for cancer treatment. First, we checked the expression of surface markers on MSC/GM-CSF since engineering could affect their recognition by the immune system. As shown in Fig. 2A, the phenotypic and immunogenicity markers were similar between MSC/GM-CSF and untransfected cells (MSC). We wondered whether modified MSCs generated via IVT mRNA transfection could result in the production and secretion of functional GM-CSF. We confirmed GM-CSF expression in the culture supernatants (conditioned media, CM) of MSC/GM-CSF by ELISA, and we detected GM-CSF protein levels up to 72 h post transfection (Fig. 2B). These results verify what we previously observed with DsRed and show that GM-CSF can be produced and secreted properly. Next, we asked whether the GM-CSF produced was functional. It has been shown that GM-CSF is involved in various cellular responses involving APCs, being one of the main functions to promote dendritic cell (DC) differentiation and proliferation [39]. To assess these functions, murine splenocytes were cultured with ConA plus CM from MSC/GM-CSF. As shown in Fig. 2C, the proliferation of splenocytes was increased when cultured with the CM from MSC/GM-CSF compared with the CM from MSC/DsRed or RPMI (control). Second, we wondered whether CM from MSC/GM-CSF was able to induce the differentiation of bone marrow progenitors into DC. Remarkably, we found that bone marrow progenitors cultured with CM from MSC/GM-CSF but not from MSC/DsRed increased the expression of CD11c^+^/CD86^+^/MHCII^+^ (Fig. 2D). LPS, a known stimulator of DC maturation, significantly increased the percentage of CD11c^+^/CD86^+^/MHCII^+^ cells in combination with CM from MSCs/GM-CSF. These results suggested that the GM-CSF produced in the engineered MSCs was able to induce appropriate DC differentiation and maturation. Next, we evaluated the effect of CM from MSC/GM-CSF on a murine cell line of macrophages (J774 cells). Notably, we found an increase in the mRNA expression of TNF-α and IL-1β after treatment with CM from MSC/GM-CSF, suggesting that the GM-CSF produced by MSCs was able to induce a macrophage switch toward a proinflammatory profile (Fig. 2E). Taken together, these results demonstrated that MSC/GM-CSF can produce GM-CSF properly and maintaining its functionality.

Then, we evaluated the therapeutic effect of MSC/GM-CSF on both HCC (Hepa129 cells) and colorectal carcinoma (CT-26 cells) tumours developed in murine immunocompetent mice. This strategy allows to study the impact of our treatment in the immune system. It has been previously reported that doses between 0.04 and 0.3 µg of cell-based GM-CSF secreting vaccine induced antitumour immune response in HCC [40]. Taking this into account, we decided to use a dose of 2 × 10^5^ MSC/GM-CSF that can produce up to 1.5 µg/ml. The cells were applied to Hepa129 or CT-26 tumour-bearing mice by peritumoral injection, and one dose was plated to further measure the GM-CSF concentration in the supernatant. Interestingly, we found that mice treated with MSC/GM-CSF exhibited significantly reduced tumour growth in both mouse models (Fig. 2F). In addition, the concentration of GM-CSF in the supernatants of 2 × 10^5^ MSC/GM-CSF was 1.48 µg/ml at 24 h. These results indicate that modified MSCs are able to produce enough functional GM-CSF that, in vivo, induced a significant reduction in tumour volume in two models of gastrointestinal tumours.

### Synergistic inhibition of tumour growth by combination treatment with MSC/GM-CSF and a low dose of doxorubicin

**Fig. 3:**
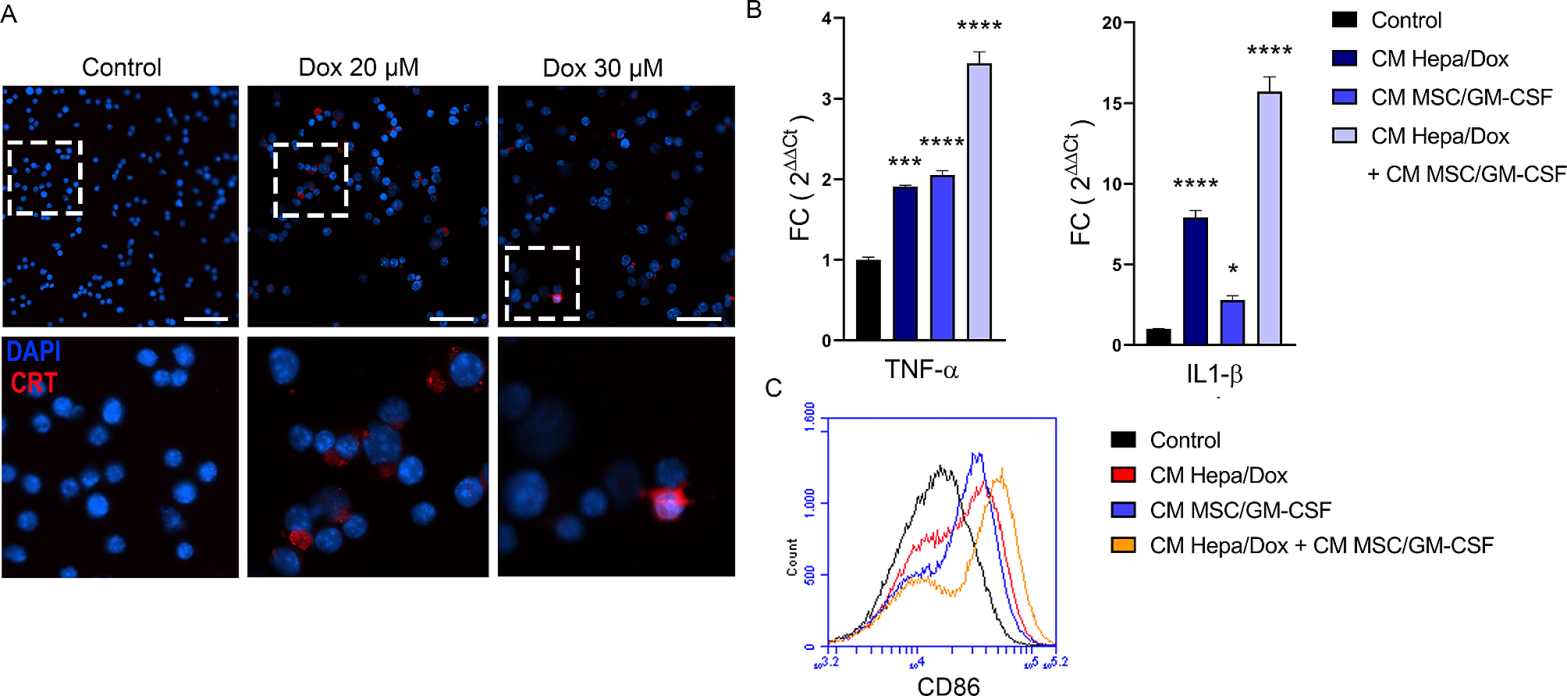
Effects of MSC/GM-CSF combined with a low dose of Dox. **A** Immunofluorescence of calreticulin (CRT, red) in Hepa129 cells after incubation for 48 h with 20 or 30 µM Dox or without treatment (control). Scale bar, 100 μm. **B** Analysis of TNF-α and IL-1β mRNA expression in J774 cells after 30 h of incubation with CM Hepa/Dox, CM MSC/GM-CSF, CM Hepa/Dox + CM MSC/GM-CSF or RPMI as control. ANOVA and Tukey’s post test, *p < 0,05, ***p < 0,0001 vs. control. **C** Representative flow cytometry image showing CD86 expression in J774 cells after 30 h of incubation with CM Hepa/Dox, CM MSC/GM-CSF, CM Hepa/Dox + CM MSC/GM-CSF or RPMI as control

**Fig. 4:**
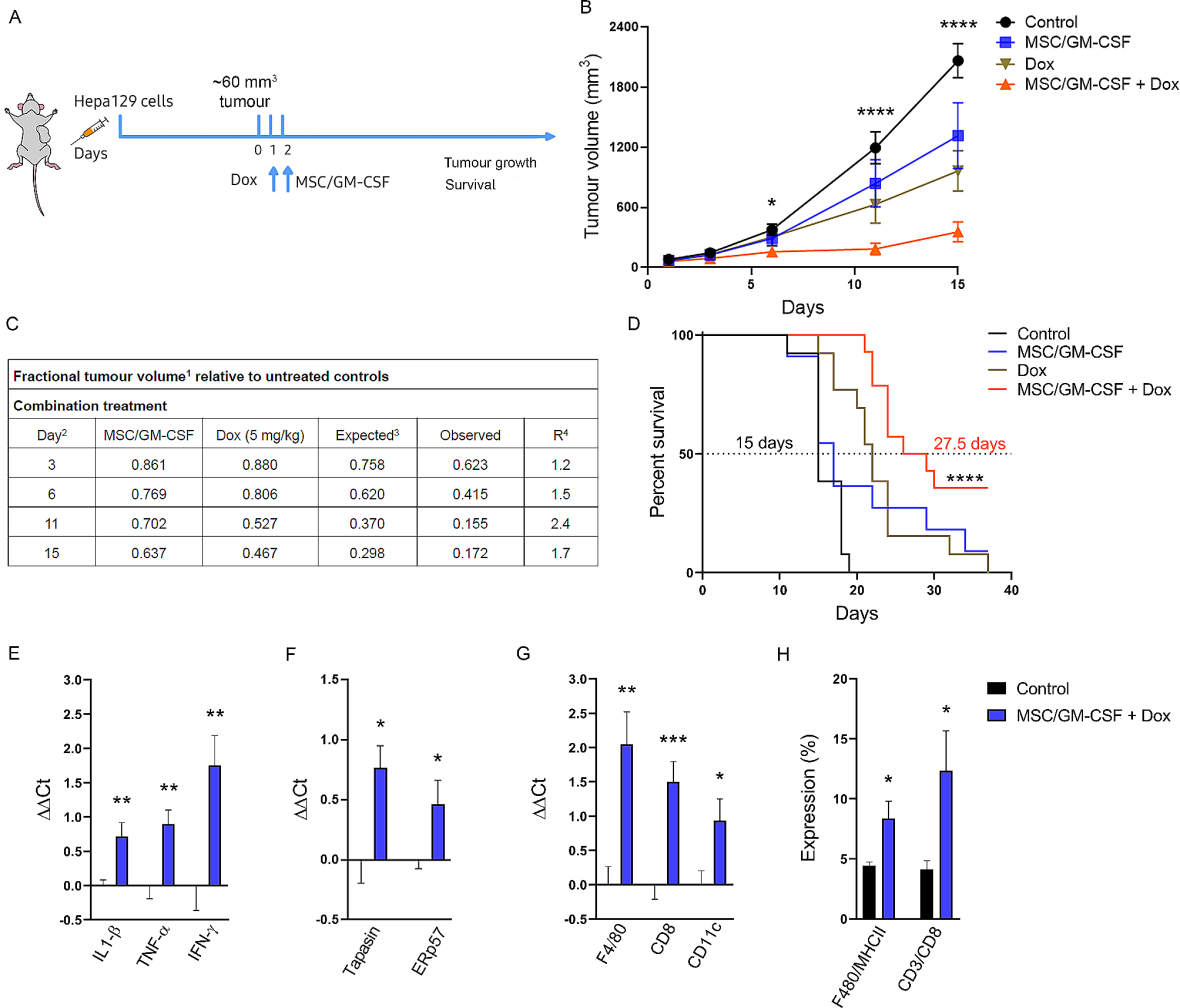
Synergistic inhibition of tumour growth by combination treatment of MSC/GM-CSF with a low dose of Dox. **A** Scheme of treatment for in vivo experiments. C3H/HeN mice were subcutaneously (s.c.) injected with 1 × 10^6^ syngeneic Hepa129 cells, and when the tumour reached ~60 mm^3^ (day 0), mice received Dox (5 mg/kg, day 1), 2 × 10^5^ MSC/GM-CSF (day2), both treatments (MSC/GM-CSF + Dox) or saline (control). **B** Tumour growth of Hepa129 tumour-bearing mice. Two-way ANOVA, *p < 0.05 and ****p < 0.0001 vs. control group. **C** Analysis of the in vivo interaction between MSC/GM-CSF and Dox by the fractional product method (FTV) in the HCC model. ^1^FTV (experimental mean tumour volume) / (control mean tumour volume); ^2^Day after treatment onset; ^3^(MSC/GM mean FTV) x (Dox mean FTV); ^4^R = [Expected FTV/Observed FTV]. A ratio > 1 indicates a synergistic effect, and a ratio < 1 indicates a less than additive effect. **D** Survival Kaplan-Meier curve, ****p < 0.0001 vs. control (log rank test). Analysis of mRNA expression of IL-1β, TNF-α and IFN-γ (**E**), tapasin and ERp57 (**F**) or F4/80, CD8 and CD11c (**G**) in mouse tumours 7 days after combination treatment (MSC/GM-CSF + Dox) or vehicle administration (control). Unpaired t test, *p < 0.05, **p < 0.01 and ***p < 0.001 vs. control. **H** Quantification of F4/80^+^/MHCII^+^ and CD3^+^/CD8^+^ cells by flow cytometry. Unpaired t test, *p < 0.05 vs. control

Recently, it has been reported that low doses of doxorubicin (Dox) induce immunogenic cell death (ICD) in cancer cells [41]. This effect is defined as a type of regulated death in which an adaptive antitumour immune response is activated through the release of damage-associated molecular patterns (DAMPs), such as the exposure of calreticulin (CRT) on the cell surface, ATP release and high mobility group box 1 (HMGB1) secretion [42]. Considering that CD8 + T cells are the primary mediators of anticancer immunity and that modulation of the CD8 + T cell response depends on APC [43], we evaluated the effect of Dox in combination with MSC/GM-CSF with the aim of increasing the observed tumour suppression. For this purpose, we first demonstrated that treatment with Dox could induce ICD in Hepa129 cells. Using immunofluorescence, we showed that in vitro CRT exposure is induced by 20 and 30 µM Dox (Fig. 3A). Next, we investigated whether DAMPs released by Dox after ICD on Hepa129 cells could enhance the proinflammatory profile of macrophages observed above by the CM from MSC/GM-CSF (Fig. 2D). To test this hypothesis, J774 macrophages were stimulated with conditioned medium from Hepa129 cells previously treated with 30 µM Dox (CM Hepa/Dox), CM from MSC/GM-CSF (CM MSC/GM-CSF), or a combination of both, after which the expression of TNF-α and IL-1β were evaluated. As shown in Fig. 3B, J774 cells treated with CM from Hepa/Dox plus CM from MSC/GM-CSF expressed higher levels of both proinflammatory cytokines than did those treated with the single treatments. In addition, by flow cytometry, we demonstrated that the maturation marker CD86 was slightly increased in macrophages after incubation with CM from Hepa/Dox or CM from MSC/GM-CSF, and the greatest increase was observed when the macrophages were treated with the combination of both CM (Fig. 3C). These results suggested that Dox-treated cells release proinflammatory signals that synergize with the GM-CSF secreted by modified MSCs. To further evaluate the effect of MSC/GM-CSF plus Dox in vivo, an HCC murine model was generated by inoculation of Hepa129 cells, and when the tumours reached ~60 mm^3^, the mice were treated with Dox (5 mg/kg) to induce ICD and DAMP release. The next day, the mice were inoculated with MSC/GM-CSF (Fig. 4A). Strikingly, compared with vehicle-treated or single-treated mice, mice that were treated with the combination therapy exhibited significant reductions in tumour progression (Fig. 4B). Remarkably, analysis of the in vivo interaction showed that while it took an average of 5–6 days for tumours to reach 300 mm^3^ in the vehicle or individual treatments, it took 14 days for tumours in animals receiving the combination therapy to reach this size. Therefore, strong synergy between treatments was revealed by a large dose enhancement factor (DEF) of 8 for the time needed to reach a volume of 300 mm^3^. In addition, this synergy is also observed in the analysis by the fractional product method (FTV) [38]. Figure 4C summarizes the relative tumour volume of the different groups at 4 different time points. On day 3 after treatment, in the combination therapy group, there was a 1.2-fold improvement in the antitumour efficacy compared to the expected additive effect. On day 11, the combination therapy group showed a 2.4-fold increase in the inhibition of tumour growth compared with that in the control group (expected fractional tumour volume). These effects were accompanied by an increase in the overall survival of tumour bearing mice after receiving the combination treatment: mice treated with MSC/GM-CSF + Dox had a sustained antitumour effect, with a median survival of 27.5 days compared to 15 days in the vehicle-treated cohort, 17 days with MSC/GM-CSF, or 22 days in the Dox group (Fig. 4D). Moreover, at the end of the experiment (day 37), 5 of the 14 mice that received the combination therapy were still alive, while only one mouse that received MSC/GM-CSF was alive among the other groups.

### MSC/GM-CSF combined with a low dose of Dox increased the antitumoural immune response

Finally, the intratumoural mRNA levels of cytokines involved in the cytotoxic response (IL-1β, TNF-α, and IFN-γ) and proteins involved in antigen presentation (tapasin and ERp57) were investigated. We found a significant increase in the levels of all the cytokines in the tumours of the mice treated with the combination treatment compared to those in the controls (Fig. 4E). On the other hand, ERp57 forms a complex with CRT that is exposed on the cell surface, providing an ‘eat me’ signal to promote phagocytosis by DCs. An increase in the mRNA expression of ERp57 in tumour samples from mice treated with the combination therapy compared to those from the control group was demonstrated (Fig. 4F). In addition, we found an increase in the mRNA expression of tapasin, which is involved in the interaction between newly assembled major histocompatibility complex class I (MHCI) molecules and the transporter associated with antigen processing (TAP), which is required for the transport of antigenic peptides (Fig. 4F). Notably, we also found increased mRNA levels of CD8, F4/80 and CD11c in tumour samples from mice treated with the combination therapy compared to the control group (Fig. 4G). Flow cytometry also revealed an increase in F4/80^+^/MHCII^+^ and CD3^+^/CD8^+^ cells 6 days after the combination treatment (Fig. 4H). Taken together, our results suggest that combination treatment with MSC/GM-CSF + Dox induces an immune response mainly through an increase in antigen presentation.

## Discussion

In recent years, several therapeutic strategies have been designed using modified MSCs as gene delivery tools. In most cases, plasmid transfection or viral vectors such as retroviruses [44], lentiviruses [45], adenoviruses [46] or adeno-associated viruses (AAVs) [47] have been used to successfully modify the gene expression of MSCs. However, the major drawback of using DNA-based vectors is their oncogenic potential due to the high risk of insertional mutagenesis. In this scenario, the use of strategies based on the transfection of IVT mRNAs will not only avoid this problem but also have high efficiency. RNA-based expression methods have great potential for use either as vaccines or for therapeutic purposes in regenerative medicine and cancer treatment [48–51]. Modified MSCs with IVT mRNA for therapeutic approaches have been tested only for glioma treatment [52] and for the delivery of immunomodulatory factors to overcome inflammation [53]. In the present work, for the first time, we propose and report the use of IVT mRNA as an alternative strategy to obtain modified MSCs for therapeutic purposes in HCC. Here, we synthesized two different IVT mRNAs that encode a reporter protein (DsRed) and a growth factor (GM-CSF) and demonstrated that their transfection into MSCs did not affect cells’ hallmarks: MSCs modified with IVT mRNA maintain their phenotypic properties, including the absence of immunogenicity markers and the ability to migrate and engraft into tumours, which are beneficial properties for therapeutic purposes in cancer treatment.

One of the main obstacles to designing IVT mRNA constructs for RNA-based therapies is high mRNA turnover in living cells. We used several strategies that have been demonstrated to increase the half-life of IVT mRNAs, including the addition of a poly(A) tail to the 3′-end, the addition of a synthetic cap analog (ARCA, 3´-O-Me-m7G(5´)ppp(5´)G) to the 5’-end and the incorporation of modified nucleosides such as 5-methylcytidine (m5C) and pseudouridine (Ψ) [22, 23]. Remarkably, we found that MSCs transfected with DsRed IVT mRNA expressed the reporter protein as quickly as 3 h and up to 15 days. It is important to note that another transient protein expression method as AAVs, requires incubation for several days for proper protein expression, whereas with IVT mRNA only a few hours. This major advantage allows a biologically safe treatment to be carried out on the same day of transfection.

Our work is the first to show that MSCs modified with IVT mRNA could be used to stimulate an antitumour response in HCC taking advantage of the properties of MSCs. It is known that MSCs play complex roles in the tumour environment depending on the MSC source and the type of tumour [54]. Even though the antitumour effect of MSCs isolated from the human umbilical cord has been tested both in vitro and in vivo in various cancer cells, such as bladder, breast, and melanoma cells, with promising results [10], our previous reports demonstrated that MSCs isolated from human umbilical cord perivascular cells did not modify HCC growth [10]. For that reason, we tested the ability of MSCs to express GM-CSF as an immunotherapeutic strategy in a subcutaneous model of HCC and observed a significant decrease in tumour growth. We also tested MSC/GM-CSF in a colorectal carcinoma murine model finding a significant decrease in tumour growth, suggesting that MSCs expressing GM-CSF could be used in other gastrointestinal tumours. In this work we injected the MSCs adjacent to tumours (peritumoural injection), but in the future, systemic application (intravenous injection) could be tested, probably by prestimulating MSCs with autocrine motility factor (AMF), a treatment that we have previously demonstrated to increase MSC migration towards HCC [55].

Immunotherapies have demonstrated great promise in the treatment of a variety of cancers. In this regard, the main strategy to improve the antitumor response in HCC involve the modulation of immunity, through the inhibition of immune checkpoints by the use of monoclonal antibodies directed against molecules such as PD-1 or PD-L1, which enables the activation of the adaptive immune response. In the last years, the block of PD-1/PD-L1 interaction alone or in combination with systemic therapy has become the first-line treatment for advanced HCC. Nevertheless, not all patients respond to immune checkpoint blockade due to the capability of HCC to induce tolerance and immune system evasion. To overcome this scenario several attempts have been explored to make the tumour emulate an infected tissue since the immune system can respond to the presence of molecular pathogens-associated patrons through the stimulation of the same receptors triggered by molecules exposed in damaged tissues (DAMPs) [56]. In this approach, we propose the use of MSCs expressing GM-CSF, a key cytokine to stimulate the antitumoural immune response [57], potentiated by the combination with Dox, which induces DAMPs liberation leading to enhance the activation of the immune system within the tumour microenvironment. On the other hand, transarterial chemoembolization (TACE) using Dox has been the standard of treatment in patients with intermediate stage HCC for more than 15 years and is associated with a mean survival of 25 to 30 months. In addition, recent studies have explored the combination of TACE with pembrolizumab (anti-PD-1) and lenvatinib (tyrosine kinase inhibitor). This study is ongoing (NCT04246177) and excludes patients with pre-existing liver diseases. Considering that our group showed that MSCs have curative properties in fibrotic livers [58] it could be interesting to design an approach that combines MSCs that have the advantage of expressing GM-CSF through IVT mRNA and TACE, directing this strategy to those patients with HCC and underlying fibrosis.

Several approaches are based on enhancing the immunogenicity of malignant cells, a critical determinant of antitumoural immune response efficacy, and boosting immune cell populations. Considering that the role of antigen-presenting cells is critical for the development of an effective immune response, we selected GM-CSF to stimulate mainly DCs and macrophages. The clinical efficacy and safety of GM-CSF was tested, for instance, combined with radiotherapy and an anti-PD-1 in a phase II trial (ChiCTR1900026175) in patients with chemotherapy-refractory solid tumours [57]. In addition, the intratumoural application of GM-CSF has also validated as a technique for attracting and stimulating DCs; however, some works have reported that GM-CSF has immunosuppressive effects on HCC [59]. To overcome this limitation, some reports have tested the use of GM-CSF in combination with IL-12 or the GM-CSF sequence inserted into an oncolytic virus vector to achieve synergistic antitumour effects on HCC models [60, 61]. Despite its beneficial effect, the dose of GM-CSF that reaches the HCC microenvironment determines its efficacy. With respect to the viral vectors, the dosage of GM-CSF could be more difficult to determine. However, using IVT mRNAs, we verified that the production of GM-CSF by MSCs was within a specific range (1,48–2.09 µg/ml 2 × 10^5^ MSCs), leading to significant inhibition of tumour growth in gastrointestinal tumour models. It should be noted that the relevance of control the expression levels of GM-CSF is required to obtain a proper therapeutic effect as demonstrated by Chen et al. [40].

In recent years, certain chemotherapeutic drugs, such as anthracyclines (Dox), have been demonstrated to trigger immunogenic cell death (ICD). After treatment with these drugs, cancer cells release DAMPs, which include HMGB1 from the nucleus, the translocation of CRT from the endoplasmic reticulum to the cell surface, and ATP secreted into the extracellular medium, promoting their recognition by immune cells [62]. In this sense, the administration of agents that can promote cancer cell damage and their elimination via ICD could be a new strategy for enhancing the efficacy of cancer treatments. Moreover, dying malignant cells, which are eliminated by T cells, initiate a tumour-specific immune response that can recognize live cancer cells [63]. This antitumour response could subsequently induce long-term clinical benefits in patients initiated by cytotoxic chemotherapy and improved by the immune arm [64]. Particularly for HCC, recent studies have demonstrated that available immunotherapies can be improved through the modulation of cells of the innate immune system, such as neutrophils and macrophages [65, 66]. Considering these findings, we used low doses of doxorubicin to potentiate the effect of MSC/GM-CSF. In this work, we demonstrated that CM from the Hepa129 cell line previously treated with Dox had an in vitro potent effect on the J774 cell line of macrophages, increasing the mRNA levels of proinflammatory cytokines and the expression of the costimulatory molecules CD80 and CD86. Tumour-associated macrophages (TAMs) are among the most abundant immune cells infiltrating the tumour microenvironment and are present at all stages of liver cancer progression [67]; therefore, our therapeutic approach takes advantage of the presence of these TAMs and stimulates them to develop a proinflammatory profile. Then, in our in vivo model, we demonstrated that low doses of Dox in combination with MSC/GM-CSF increased the number of macrophages (F4/80^+^), the number of cytotoxic T cells (CD3^+^/CD8^+^) and the levels of proinflammatory cytokines within the tumour. Furthermore, we found an increase in the mRNA levels of two proteins involved in peptide loading into the MHCI and an increase of the CD11c marker in animals treated with the combination treatment of MSC/GM-CSF + Dox. These results suggest that our strategy could favour the assembly of a specific response, considering that the function of MHCI is to present fragments of proteins produced inside cells to T lymphocytes and subsequently develop a specific response, although additional experiments are needed to confirm this phenomenon. In this work, we used low doses of Dox in combination with MSC/GM-CSF to increase the amount of DAMPs in the tumour, decreasing the proinflammatory profile of TAMs. In our study, we found a remarkable antitumour effect of the combination of the ICD strategy through low doses of Dox with the application of MSC/GM-CSF in an immunocompetent murine HCC model. Although the murine model used was chosen due to the intrinsic similarity of the murine immune system with the human one, more studies are needed to prove the efficacy observed in our study could be translated to humans.

In conclusion, we provide strong evidence that the secretion of GM-CSF by IVT mRNA-modified MSCs in combination with low doses of Dox significantly decreases HCC growth, indicating that this approach exhibits strong synergistic effects and could be a new powerful tool for improving current cancer treatment strategies.

### Electronic supplementary material

Below is the link to the electronic supplementary material.

Supplementary Material 1

## Data Availability

All materials and data can be available in the Manuscript and Additional file.
