# Supplementary material for: Modified mesenchymal stromal cells by in vitro transcribed mRNA: a therapeutic strategy for hepatocellular carcinoma
Source: Stem Cell Res Ther. 2024 Jul 11;15:208. doi: 10.1186/s13287-024-03806-0 (PMC11241816; doi:10.1186/s13287-024-03806-0)
Supplement: Supplementary file 1 — Supplementary Material 1 [file 13287_2024_3806_MOESM1_ESM.docx]

**Supplementary Table 1.** Primer sequences for the design of IVT mRNAs

| **GENE** | **PRIMER** | **SEQUENCE** |
| --- | --- | --- |
| mGM-CSF | Forward | 5´-TTA TCG AAA TTA ATA CGA CTC ACT ATA GGG-3´ |
|  | Reverse | 5'- TTTTTTTTTTTTTTTTTTTTTTTTTTTTTTTTTTTTTTTTTTTTTTTTTTTTTTTTTTTTTTT  TTTTTTTTTTTTTTTTTTTTTTTTTTTTTTTTTTTTTTTTTTTTTTTTTTTTTTTTTCTGGATTCAGAGCTGGCCTG -3´ |
| DsRed | Forward | 5'- CCGACTTAATACGACTCACTATAGGGCGATAATACCATGGCCTCCTCC -3' |
|  | Reverse | 5'- TTTTTTTTTTTTTTTTTTTTTTTTTTTTTTTTTTTTTTTTTTTTTTTTTTTTTTTTTTTTTTTTT  TTTTTTTTTTTTTTTTTTTTTTTTTTTTTTTTTTTTTTTTTTTTTTTTTTTTTTTCTACAGCCAGGGCCAGGAGAG -3' |

**Supplementary Table 2.** Primer sequences for qPCR

| Gene | Forward | Reverse |
| --- | --- | --- |
| TNF-α | 5′-GACCCTCACACTCAGATCATCTTCT-3′ | 5′-CCACTTGGTGTTTTGCTA CGA-3′ |
| F4/80 | 5′-CTTTGGCTATGGGCTTCCAGTC-3’ | 5′-GCAAGGAGGACAGAGTTTATCGTG-3’ |
| IL-1β | 5′-TGACAGTGATGAGAATGACCT GTTC-3’ | 5′-TTGGAAGCAGCCCTTCATCT-3′ |
| GAPDH | 5′-GGGGCTGCCCAGAACATCAT-3’ | 5′-GCCTGCTTCACCACCTTCTTG -3′ |
| CD8 | 5’-CCGTTGACCCGCTTTCTGT-3’ | 5’-CGGCGTCCATTTTCTTTGGAA-3’ |
| F4/80 | 5’-CTTTGGCTATGGGCTTCCAGTC-3’ | 5’-GCAAGGAGGACAGAGTTTATCGTG-3’ |
| ERp57 | 5’-ATGCGCTTCAGCTGCCTAG-3’ | 5’-AGAACTCGACCAGCATGAGC-3’ |
| Tapasin | 5’-CTGGTTCGTGGAGGATGCAG-3’ | 5’-GCTTTGGGTCAAGATCTGGC-3’ |
